# In-depth comparison of two quality improvement collaboratives from different healthcare areas based on registry data—possible factors contributing to sustained improvement in outcomes beyond the project time

**DOI:** 10.1186/s13012-019-0926-y

**Published:** 2019-07-23

**Authors:** Beatrix Algurén, Annika Nordin, Boel Andersson-Gäre, Anette Peterson

**Affiliations:** 1 Department of Food and Nutrition, and Sport Science, University of Gothenburg, Faculty of Education, Box 300, 405 30 Gothenburg, Sweden; 2 School of Health and Welfare, Jönköping Academy for Improvement of Health and Welfare, Jönköping University, Jönköping, Sweden; 3 Futurum, Region Jönköping County, Jönköping, Sweden

**Keywords:** Collaboratives, Quality improvement, Teams, Learning, Measurement skills, Data warehouses

## Abstract

**Background:**

Quality improvement collaboratives (QICs) are widely used to improve healthcare, but there are few studies of long-term sustained improved outcomes, and inconsistent evidence about what factors contribute to success. The aim of the study was to open the black box of QICs and compare characteristics and activities in detail of two differing QICs in relation to their changed outcomes from baseline and the following 3 years.

**Methods:**

Final reports of two QICs—one on heart failure care with five teams, and one on osteoarthritis care with seven teams, including detailed descriptions of improvement projects from each QIC’s team, were analysed and coded by 18 QIC characteristics and four team characteristics. Goal variables from each team routinely collected within the Swedish Heart Failure Registry (SwedeHF) and the Better Management of Patients with OsteoArthritis Registry (BOA) at year 2013 (baseline), 2014, 2015 and 2016 were analysed with univariate statistics.

**Results:**

The two QICs differed greatly in design. The SwedeHF-QIC involved eight experts and ran for 12 months, whereas the BOA-QIC engaged three experts and ran for 6 months. There were about twice as many activities in the SwedeHF-QIC as in the BOA-QIC and they ranged from standardisation of team coordination to better information and structured follow-ups. The outcome results were heterogeneous within teams and across teams and QICs. Both QICs were highly appreciated by the participants and contributed to their learning, e.g. of improvement methods; however, several teams had already reached goal values when the QICs were launched in 2013.

**Conclusions:**

Even though many QI activities were carried out, it was difficult to see sustained improvements on outcomes. Outcomes as specific measurable aspects of care in need of improvement should be chosen carefully. Activities focusing on adherence to standard care programmes and on increased follow-up of patients seemed to lead to more long-lasting improvements. Although earlier studies showed that data follow-up and measurement skills as well as well-functioning data warehouses contribute to sustained improvements, the present registries’ functionality and QICs at this time did not support those aspects sufficiently. Further studies on QICs and their impact on improvement beyond the project time should investigate the effect of those elements in particular.

## Introduction

Worldwide, delivering safe healthcare of high quality remains challenging. Health care leaders and policymakers strive to develop effective strategies and methods to support providers in improving practices and thereby patient outcomes. In recent years, quality improvement collaborative (QIC) methods have become popular for driving improvements in the quality of care and implementation of evidence-based practices [1]. A QIC can be defined as a structured approach for improvement built on joint learning and improvement with teams from multiple organisations. A QIC has five essential features: (a) it has a specified topic; (b) it combines clinical experts and experts in quality improvement (QI) who provides knowledge, ideas and support for improvement; (c) it includes multi-professional teams from multiple sites; (d) and a model for improvement (setting targets, collecting data and testing changes); and (e) the collaborative process involves a series of structured activities [2–5].

Earlier studies on the effect of QICs (including literature reviews) have shown variation in results, not to mention little focus on long-lasting effects. Certain QIC components affected the QIC outcomes unequally when compared across different QICs. For example, in a study from Gustafson et al., coaching was shown to be the most effective way to reduce waiting time and to increase the number of new patients in addiction treatment services across 201 clinics, but it had no effect on retention rate [6]. While in the latter study, teleconferences and learning sessions had no significant effect on any of the three outcomes, these components were identified as enhancing the short-term success of 26 QICs in a systematic review from Hulscher et al. [7]. However, in that review, 80% of 121 eligible papers lacked quantitative tests of possible success determinants and thus had to be excluded [7]. Studies have been hard to interpret and compare, since QIC components have often been insufficiently described, or used unevenly [8, 9]. Among the 14 crosscutting QIC components identified in a review (e.g. in-person learning sessions with training in QI methods, online meetings, data reporting and leadership involvement), on average only half of these components were included in QICs [8]. Furthermore, a paucity of detailed descriptions of QIC components (e.g. the quality improvement process itself, the use of data and feedback, intensity of QIC approach) was noticed by two literature reviews [8, 10]. The lack of detailed descriptions that would make either scientific or practical reproduction possible was identified as a problem not only for QICs but also for implementation strategies in general [11, 12]. Additional research on the success of QICs and their impact on patient care should therefore reveal ‘the black box’ with a detailed description of activities but also with more data on outcomes in order to allow generalisation and make findings from one healthcare area useful to another [8, 10]. Moreover, in the area of eHealth, the nature of QICs might be revolutionised by technological advances in electronical health records and by well-functioning clinical registries with available and timely feedback loops.

In Sweden, clinical registries have a long tradition starting in the 1970s with the Swedish Knee and Hip Arthroplasty Registers which monitor the success of arthroplasty. These Swedish National Quality Registries (NQRs) contain ‘individualised data concerning patient problems, medical interventions, and outcomes after treatment; within healthcare services.’ (www.kvalitetsregister.se). They are in many respects valuable tools for improving healthcare quality in Sweden [13–17]. During the last decade, several QICs have been carried out using NQRs, leading to good results in improved adherence to national guidelines [18–20]. A recent study of two cardiovascular NQRs showed, however, that they were infrequently integrated into clinical practice and or continuous quality improvement activities; most of the users reviewed the collected data less than three times per year [21]. Moreover, little attention has been given to the long-term effects of QICs and how improved outcomes are sustained over time [8]. Since ‘a collaborative is a complex and time-consuming intervention for clinicians, teams and sites and represents major financial, organisational and political investment’ (p. 235, [10]), it is important to get a better understanding of how QICs work and what QIC components are essential for short- and long-lasting effects with regard to the heterogeneous healthcare microsystems and contexts.

The present study intends to contribute to a deepened understanding of the generic components of QICs and team activities which are associated with sustained outcomes beyond the project time and across the different healthcare areas and contexts.

The aim of the present study is to compare 12 teams from two QICs using NQRs from different areas of healthcare—heart failure and osteoarthritis. More precisely, we have described in detail and compared (a) the components of the respective QIC, (b) the characteristics and activities of each QIC team and (c) the longitudinal outcomes over 3 years after the launch of the QICs. In particular, we studied the differences between the QICs and between the teams and their activities, how they are linked to each other and how they are inter-related with their longitudinal outcomes.

## Methods

### Study background

This study is part of a larger project adopting a mixed-methods approach with the objective of studying the effects of a government-funded 5-year national program on enhancing the use of NQRs in Sweden. In Sweden, health and social care is financed by taxes. While healthcare is administered by 20 county councils and regions, social services and in-home healthcare are managed by 290 municipalities. NQRs were identified to be insufficiently used for improvement and research, but could have a great potential to support improvement quality in healthcare [22]. In order to enhance use of NQRs for quality improvement in healthcare, the government and Swedish Association of Local Authorities and Regions (SALAR) committed to increase the funding of NQRs between 2012 and 2016 [23]. Within the scope of this initiative, so-called quality registry centres were established and/or further developed if already existing for each of the six healthcare regions with the aim of supporting the management and utilisation of NQRs.

### Study design and sample

The present case study had a multiple-case embedded design, e.g. multiple units of analysis [24]. The multiple cases were two QICs initiated by the registry centres and aiming to increase the use of NQRs for quality improvement. The multiple embedded units of analysis were the participating teams in each QIC. The selection of cases was based on purposive sampling and represented different kinds of registries from two different areas of healthcare. Multiple case studies have the advantage that the evidence created by them is seen to be stronger and more reliable compared to single case studies [24]. By comparing the different cases—which in the present study were the two QICs from different healthcare areas with a total of 12 teams—contrasts and similarities across situations could be discussed while the different contexts were considered, and suggestions were grounded in several empirical evidence [25]. One QIC was chosen within heart failure care using the Swedish Heart Failure Registry (SwedeHF) and one QIC within osteoarthritis care using the Better Management of Patients with OsteoArthritis Registry (BOA).

The BOA was established in 2008, while the SwedeHF was established in 2003. End-users of the BOA are physiotherapists, whereas end-users of the SwedeHF are mainly physicians, but also nurses. The BOA aims to evaluate patient-reported outcomes following an intervention—the Supported Osteoarthritis Self-Management Programme (SOASP). Information collected in the BOA helps to monitor the outcomes of physiotherapists’ or occupational therapists’ interventions on patient level (https://boa.registercentrum.se/boa-in-english/better-management-of-patients-with-osteoarthritis-boa/p/By_o8GxVg). The SwedeHF aims to standardise and monitor the diagnosis and treatment of patients with heart failure, in order to ensure equal and high-quality care for patients nationally. The information given in the SwedeHF guides physicians and facilitates optimal management of patients with heart failure (www.ucr.uu.se).

Both the SwedeHF and the BOA collect longitudinal data, at first registration and at follow-up, 1 year later. The BOA also has one follow-up after 3 months. Whereas the BOA comprises 93 variables with mainly patient-reported outcome measures (50%), the SwedeHF comprises 163 variables, mainly clinician-reported outcome measures, and process and administrative data (30%, 35% and 25%) as distinguished by Donabedian’s quality criteria [26].

### Data collection

For the present study, two sources of information were used: (a) the final project report of each QIC, and (b) registry data from 2013 to 2016 each year from the BOA and the SwedeHF respectively. The final project reports did not have a standardised structure but both contained information on the QIC itself and described activities and learning for each team. The report from the SwedeHF-QIC also contained posters of each team summarising their aims, methods, results and conclusions from their individual project within the QIC. If there was missing information, the respective project leader was contacted and asked for the information.

Out of the many variables routinely collected in each registry, only the small quantity of data was surveyed that was addressed by the QIC as concerned with goal variables. These variables were monitored on a yearly basis from the baseline in 2013 when the QIC projects started, to 2016, 2 years after the QIC projects had finished. For the BOA, the following outcomes were extracted: (1) the percentage of completed 3-month follow-ups, (2) the average age of registered patients and (3) the percentage of patients with a minimum level of physical activity at the three-month follow-up. For the SwedeHF, the following outcomes were obtained: (1) the percentage of patients with left ventricle measurement, (2) the percentage of eligible patients receiving beta-blockers and renin-angiotensin system (RAS) treatment, (3) the percentage of patients with planned follow-up and (4) the percentage of patients undertaking organised physical activity. Additionally, for both QICs, the number of registered patients was collected. Since the total number of persons with heart failure or osteoarthritis in each region might differ, it is not the total amount that shows how appropriate teams worked but rather teams’ work load for the respective patient group. Each registry-specific data-set was obtained from the coordinator of the respective registry.

### Data analysis

Analysis was conducted using both quantitative and qualitative methods. The final project reports were analysed by content analysis and coded by (a) a coding scheme with 18 topics in order to describe QIC components, and (b) a second coding scheme with five topics in order to describe in detail the team characteristics and QIC activities. To identify the latter, a thematic analysis was used, where activities carried out by the teams were extracted from the report, sorted and grouped into themes and counted [27]. The first coding scheme was guided by the 14 crosscutting QIC components identified in the review of Nadeem et al., namely length of collaborative, convened expert panel, organisation required to demonstrate commitment, in-person learning sessions, Plan-Do-Study-Act (PDSA) circles, multidisciplinary QI team, QI team calls, email or web support, sites collected new data for QI, sites reviewed data and used feedback, external support with data synthesis and feedback, leadership involvement, training for ‘non-QI team staff members’ by experts and training for ‘non-QI members’ by the QI team [8]. This list of QIC components was considered to be the most-complete evidence on QICs based on a comprehensive literature review and expert opinion [8]. In addition to those 14 components, we added ‘project responsible’, ‘invitation/information’ (how teams were recruited), ‘overall goal of QIC’ and ‘costs’ to the coding scheme (see Table 1). The second coding scheme with five topics comprised characteristics of QI team (profession and number of participants), teams’ main goal, source of collected data for QI, data reviewed during project time and overarching themes and their number of team activities performed during the project time. All coding was done by the first author and reviewed by responsible persons of each QIC. In case of disagreement, consensus was reached and the coding was changed accordingly.

**Table 1:** Overall characteristics of the QI collaboratives structured by the 14 crosscutting QIC components (identified from Nadeem et al. by a comprehensive literature review and expert opinions [8])

	BOA-QIC	SwedeHF-QIC
1) Length of project	6 months	12 months
2) Convened expert panel Breakthrough series model calls for a planning group that identifies targets for improvement change and plans the collaborative.	3 experts (including head of register)	8 experts (one project group with five persons, and one steering group with three persons including head of register)
3) Organisations required to demonstrate commitment^a^	Yes	Yes
4) In-person learning sessions	2 days	8 days (4 × 2 days)
5) Plan-Do-Study-Act cycles (PDSAs)	Yes	Yes
6) Multidisciplinary QI Team	Not specified	Yes (patients were included)
7) Project responsible at unit	Yes	Yes (called coach)
8) QI team calls Calls among QI team members or members in other participating organisations are common.	No A mailing list with all participants was available and participants were encouraged to do so.	Yes Coaches had the task of participating in meetings with all coaches (a phone call once a month, in total 10 times).
9) Email or web Support Email, listservs, or others forms of web support have become a common approach for providing ongoing support.	Yes, Done by the head of register.	Yes, Done by the project group. 8 webinars were provided.
10) Leadership involvement/outreach	Not specified	Yes, guaranteed by the coaches
11) Sites collected reviewed data and used feedback	Yes	Yes
12) External support with data synthesis and feedback	Not specified	Yes (QI team members experienced data extraction as difficult)
13) Training for ‘non-QI Team Staff Members’ by experts	No	Yes, indirectly (8 webinars were open to everybody)
14) Training for ‘non-QI members’ by the QI team	Yes	Partly
Additional information		
Project responsible^b^	One competence centre for national quality registries (A)	Two competence centres for national quality registries (B + C)
Information/invitation	Internal to all registering units	Open on the web
Overall goal	A) Decrease of average age of registered persons (e.g. discovery of patients with osteoarthritis in an early stage) B) Increased number of patients with a minimum level of physical activity after one year	A) Better quality of life for persons with heart failure B) Decreased cases of re-admission within 30 days^c^
Costs	32 000 USD	166 000 USD

^a^Some interested teams withdrew because of non-commitment

^b^Six regional competence centres for the National Quality Registries have been established with the mission to promote development of new registries and to provide service to existing registries, for example for technical operations, analytical work and use of registry data supporting clinical quality improvement [14]

^c^Identified steps in order to reach goal: correct diagnosis, treatment recommended, structured follow-up at heart failure units, collaboration between primary care and hospital, quality evaluation by using the SwedeHF. Measurements: number of patients with control of left ventricle function is at least 90%, with RAS-blockers treatment is at least 90%, with beta-blockers treatment is at least 90%, that participated in organised physical activity is at least 90%, with structured follow-up at heart failure units is at least 90%

**Fig. 1:**
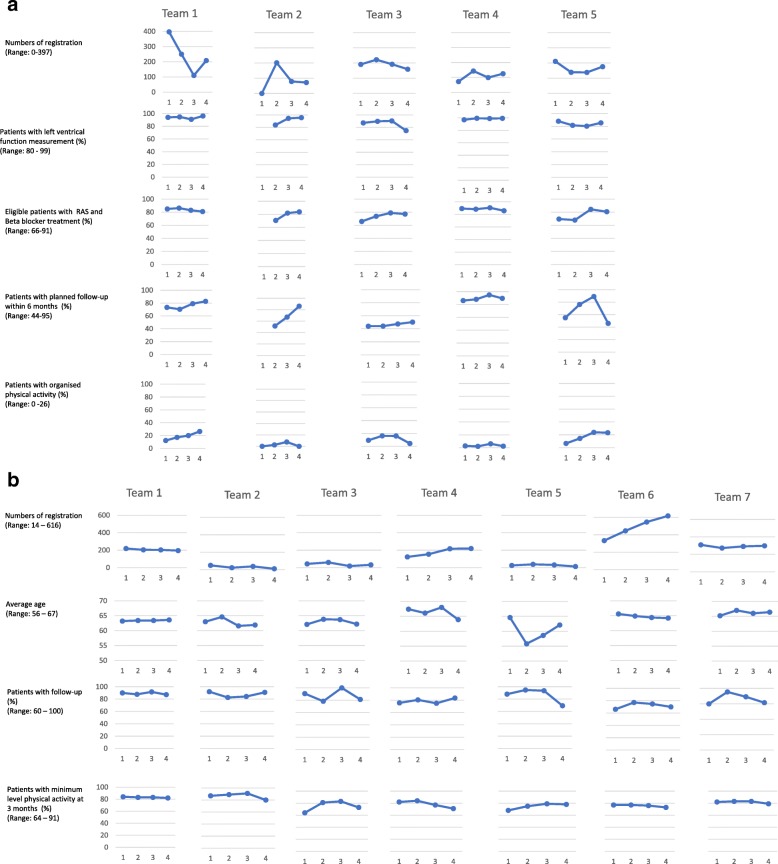
**a** Changes in the SwedeHF-QIC’s goal outcomes from baseline to 2 years post project. 1 = year 2013, 2 = 2014, 3 = 2015, 4 = 2016. **b** Changes in the BOA-QIC’s goal outcomes from baseline to two and half years post project

Registry data were analysed by univariate statistics (Excel programme, version 16.19 was used) and described with frequencies or percentage of registered patients at baseline year 2013 as well as 2014, 2015 and 2016 (see Figure 1).

### Ethical consideration

This study is based on public documents and aggregated data from NQRs on group level. Neither individual patients nor team participants can be identified. To support confidentiality, team names are replaced with numbers in the text and figures. Furthermore, the study does not focus on the actions or performance of individuals but on improvement efforts undertaken by the organisations and thus the study did not meet the criteria to require approval from a Swedish ethical review board. Nonetheless, the study has been carefully designed to safeguard any ethical issues.

## Results

### Component characteristics of the two quality improvement collaboratives

Table 1 gives an overview of the characteristics of each QIC. The two QICs differed greatly in design. SwedeHF-QIC involved eight experts and had a project time of 12 months including five teams (multi-professional including patients). The BOA-QIC engaged three experts, ran for 6 months and included seven teams. Registry holders (individuals with overall responsibility for an NQR) were included in both QICs as experts, but their roles differed. In the BOA-QIC, the registry holder led the QIC, supported by experts on quality improvement. In the SwedeHF-QIC, experts on quality improvement led the QIC, facilitated by the registry holder. The number of learning seminars (collective seminars) was more than four times higher in the SwedeHF-QIC than in the BOA-QIC (8 days versus 2). Shared characteristics of the two QICs were organisational commitment backing up participating teams, introduction to ‘plan-do-study-act circles’, designated persons responsible for improvement work at each unit, overall goals of the QICs determined by experts, ongoing email support (by registry holder, though less used in the BOA-QIC than by QI experts in the SwedeHF-QIC) and review of data to identify interventions necessary in each team to improve quality.

Telephone conferences and facilitated learning between teams as well as leadership involvement, appeared in the SwedeHF-QIC through the role of coaches. Teams in the BOA-QIC reported no contact with each other between learning seminars even though this was encouraged by the provision of an email list. Leadership involvement was also not specified. Training by quality improvement experts for staff that were not members of QIC teams was possible through eight open access webinars in the SwedeHF-QIC, but did not occur in the BOA-QIC. However, training of staff by the QI team itself (e.g. not the QI experts) was done in the BOA and only partly in the SwedeHF. Overall, the costs were five times higher for the SwedeHF-QIC (166000 USD) than for the BOA-QIC, excluding travel and accommodation costs for teams participating in learning seminars [28, 29].

### The characteristics and goals of teams from the SwedeHF-QIC

Table 2 gives an overview of the characteristics of the teams and their activities performed by the SwedeHF-QIC teams. All five SwedeHF-QIC teams were multi-professional, with six members on average (median, range 3–10 members) including nurses but also physicians, healthcare quality developers and leaders. At the end of the 12 months project time, the teams finally succeeded in including patient representatives. The overall goal of each QIC was set by the expert team and was connected to the overall improvement goal of each registry within the national programme. In the case of the SwedeHF, the overall goal was to reduce re-admission rates within 30 days post-diagnosis. One team participated in order to get started with registrations into the registry. All teams strived to improve their care processes through better teamwork (increased cooperation between different professions) along the whole chain of care (increased cooperation between different care providers). Two teams also included ‘correctly diagnosing and better identifying patients at risk’ as an aim.

**Table 2:** Characteristics of the SwedeHF-QIC teams and their activities performed during the project time (1 year). All teams were employees of regional hospitals

QI team (Total number of participants, *n*)	Team 1 (8)	Team 2 (5)	Team 3 (8)	Team 4 (3)	Team 5 (10)
Profession of participants			^c^		
Nurse	2	4		2	8
Physician	1				1
Healthcare quality developer	2	1	1		
Leader of unit	3			1	1
Others	1 PT		1 pharmacist		
Teams’ main goal					
Improve care process^a^	x	x	x	x	x
Improve diagnosis^b^		x	x		
Others				Increased visits to PTs.	
Source of collected data for QI					
Health records (re-admission rate)	x	x	x	x	x
Registry	x	x	x	x	x
Data reviewed during project time					
At baseline (supported by QIC experts)	x	x	x	x	x
Ongoing					
At the end	x	x	x	x	x
Overarching themes of activities performed (*n* = number of different activities)					
Increased availability and follow-up (among others with focus on physiotherapy)	3		1	1	4
Development of and adherence to standard care programme, diagnosis and treatment guidelines	1	3			3
Standardised information about diagnoses and treatments (among others with focus on physical activity)	1			2	1
Cooperation and communication along the chain of care between different stakeholders	1	1	2		1
Participants’ perceived overall experience of QIC (information received from the final QIC report)	‘…. participating teams are satisfied with the QIC activities, and that they have learnt improvement methods ….’ p.2				
	‘The results of the surveys showed that overall, the participants appreciated our seminars. Several participants highlighted patient participation as a very good part of the project. The final project evaluation showed that all participants could recommend the programme to a colleague and that they were satisfied with the program.’ p. 6				

*PT* physiotherapist

^a^Improve care process through better team work (different professions) along the whole chain of care (different stakeholders)

^b^Improve diagnosis and identification of patients at risk

^c^Multidisciplinary from fields such as medicine, geriatric, rehabilitation, primary care, home care but not specified profession

### The characteristics and goals of teams from the BOA-QIC

Table 3 gives an overview of the BOA-QIC team characteristics and their activities. At the first learning seminar, five of seven BOA-QIC teams participated with a minimum of one physiotherapist (the median number of members was two). Two ‘teams’ were represented by a single physiotherapist. However, these physiotherapists were asked to function as project leaders at their home work units, driving the interventions forward. Additionally, co-workers at these work units were expected to engage in the work. Two BOA-QIC teams were multi-professional. In addition to the physiotherapists, the teams included physicians or heads of unit. Along the overall goal to decrease the average age of registered patients, three BOA-QIC teams aimed to increase knowledge about osteoarthritis and SOASP among professionals (mainly orthopaedists and occupational therapists) and two BOA-QIC teams aimed to increase the number of patients engaging in the recommended level of physical activity either 3 months or 1 year post SOASP. One BOA-QIC team tried to standardise their SOASP to their patient group. By reviewing registry data, another BOA-QIC team recognised that, prior to other changes, they needed to improve registration into the registry and their routines to review results monthly.

**Table 3:** Characteristics of the BOA-QIC teams and their activities performed during project time (6 months), all teams were employees in primary care

QI team (Total number of participants, *n*)	Team 1 (2)	Team 2 (2)	Team 3 (1)	Team 4 (3)	Team 5 (2)	Team 6 (1)	Team 7 (2)
Profession of participants							
Physiotherapist	1	1^a^	1^a^	3	2^a^	1^a^	2^a^
Physician		1					
Leader of unit	1						
Teams’ main goal							
Increase number of patients with recommended level of physical activity	x				x		
Increase knowledge about osteoarthritis and SOASP among health professionals		x	x	x			
Others						Standardised way of working adapted to patients’ needs	Increased number of registrations into BOA^b^
Source of collected data for QI							
Registry	x	x	x	x	x	x	x
Data reviewed during project time							
At baseline (supported by QIC experts)	x	x	x	x	x	x	x
Ongoing							
At the end							
Overarching themes of activities performed (*n* = number of different activities)							
Increased availability and follow-up	1			1	2		
Development of and adherence to guidelines and routines		1				1	
Education and information about osteoarthritis and SOAP		1	2	4			
Others							Develop routine to register patients; develop routine to review data from registry monthly
Participants’ perceived overall experience of QIC (information received from the final QIC report)	‘Everyone agreed that it was very educational and we found that despite the short project period, ambitious and targeted efforts were carried out on the units, using the new working methods that really boosted the improvement processes.’ p. 6						

^a^Participated only at introduction seminar

^b^95% of all patients who attend Supported Osteoarthritis Self-Management Programme (SOASP) should complete the questionnaire and 95% of those completed questionnaire should be registered by physiotherapists in the BOA system. Follow user manual

Whereas all teams from the SwedeHF-QIC were employees of different regional hospitals, all teams from the BOA-QIC were employees of different primary care facilities. Participants in both cases appreciated the QIC program and perceived that it contributed to their own learning about improvement methods.

### The activities of teams from the SwedeHF-QIC

On average, the SwedeHF-QIC-teams identified five activities each to improve care (see Table 2). Those team activities could be categorised and structured into four overall themes: (a) Increased availability and follow-up (among others with focus on physiotherapy) (four out of five teams); (b) Development of and adherence to a standard care programme, diagnosis and treatment guidelines (three out of five teams); (c) Standardised information about diagnoses and treatments (including a focus on physical activity) (three out of five teams); and (d) Improving cooperation and communication along the chain of care between different care providers (four out of five teams). The activities had in common that cooperation among physicians, nurses, physiotherapists as well as patients was identified as important to improve through better structure of task distribution and agreement on it among professionals and stakeholders. In particular, teams established care programmes and agreements between primary care and specialist clinics in hospitals, created better care plans and processes for follow-ups, checklists and guidelines for physicians and nurses, developed patient information sheets and established teamwork with physiotherapists.

### The activities of teams from the BOA-QIC

On average, two activities were performed by the BOA-QIC teams (see Table 3). Those activities could be categorised into three overall themes: (a) Increased availability and follow-up (three out of seven teams); (b) Development of and adherence to guidelines and routines (two out of seven teams); and (c) Education and information about osteoarthritis and SOAP (three out of seven teams). To increase availability and follow-up, teams started to offer SOAP training in the evening, introduced structured follow-up calls after 3, 6 and 9 months post SOAP, and in the case of a decreased level of physical activity, individual healthcare visits were booked. Education and information could be provided by workshops and lectures for physicians, written standardised information for patients and for health professionals and posters on care units and orthopaedic hospitals. In order to develop guidelines, team discussions on routines and on optimised care processes were held and checklists were formalised. There was consensus among participants that, despite the short running time, the QIC program contributed to their learning and ‘…. ambitious and targeted efforts were carried out on the units, using the new working methods that really boosted the improvement processes.’ (Final QIC report, p.6) [29].

### Data reviewed during QIC project time

The SwedeHF-QIC teams’ initial data collection comprised a screening of health records to obtain the re-admission rate since improvement of the latter was the overall goal. Each team included at least one person who was responsible for entering data into the registry at the unit and as such was more familiar with the system. Participants were surprised at how difficult it was to extract and follow-up this information. The registry’s system functionality impeded data extraction and longitudinal comparison at unit level. Thus, at the last learning seminar of the QIC project, all teams compared longitudinally at least one indicator on a yearly basis. The content of the other learning seminars concerned heart failure itself, improvement knowledge and methods, oral dialogues for exchange of experiences and discussions on ongoing activities as well as own work.

For the BOA-QIC, data from the registry on unit level were presented by the head of register at the first learning seminar. Each team included a person who collected data for the registry (that could be on a paper sheet). However, system functionality impeded data extraction and longitudinal comparison at unit level also for the BOA users. Thus, as well as the awareness that the QIC’s overall goal to ‘decrease average age among patients’ could not be achieved within 6 months, data were not reviewed continuously by the teams. The second and last learning seminar was about exchange experiences on activities performed, e.g. an information sheet about SOAP.

### Longitudinal results of registry data-based outcomes

Figure 1a, b shows the registry data-based outcomes of the SwedeHF-QIC and of the BOA-QIC each year between 2013 and 2016. Overall, by comparing outcomes between the years, the results were found to be heterogeneous within teams (e.g. not all goal outcomes improved, only two out of four), across teams and across the two compared cases of QICs.

All the SwedeHF-QIC teams were significantly under the goal of 90% of registered patients with organised physical activity (ranging from 2 to 26%), though all the SwedeHF-QIC teams showed improvements between 2013 and 2014, and teams one (including a physiotherapist) and five showed continuous improvement until 2016. Similarly, the goal of 90% of registered patients with structured follow-up within 6 months was difficult for the SwedeHF-QIC teams to achieve (cave: available data were ‘planned’ and not the number of patients that were truly followed up). However, the SwedeHF-QIC teams were closer to this goal than before the launch of the QIC, and two teams reached the goal in 2015 and one also in 2016 (teams 4 and 5), whereas the results of the other SwedeHF-QIC teams ranged between 44 and 86%. Two out of five teams (1 and 4) had already reached the goal of 90% of registered patients with left ventricle function measurement before they participated in the QIC. Team 2 reached this goal in 2014 and 2015, and team 3 in 2015 and 2016. All teams reviewed the change of re-admission rate within 30 days at the end of QIC in 2014 with data from health records.

Regarding the BOA-QIC, one team reached the goal of decreasing the average age of registered patients to 58 years over 2 years (2014 and 2015, team 5). The goal that 80% of registered patients should be physically active at a defined minimum level was reached by three BOA-QIC teams for all years (teams 1, 2 and 7) and at times was attained by the other teams. Most of them maintained the values continuously, with small variations.

### Comparison between QICs’ characteristics, activities and their longitudinal results

A clear relationship between the characteristics of the QICs (such as the number of participants, their role, number of learning sessions) and the longitudinal results on outcomes could not be seen, but the longer project time in the SwedeHF-QIC supported a higher number of interventions than in the BOA-QIC (five interventions versus two on average). Improvements tended to continue if teams’ activities included structural change such as improved guidelines (central and local) or common care programmes (SwedeHF-QIC teams 2 and 3). Also, including a physiotherapist in the team had positive long-term impact on increased physical activity among heart failure, even improving years after the QIC (team 1). Increased availability and follow-up, development of and adherence to guidelines and standard care programmes, as well as standardised information about diagnosis and treatments were three common activities that both QICs worked with in order to improve and reach their goals.

## Discussion

This study described two QICs with five versus seven teams in each, their activities and their longitudinal outcomes. Despite the fact that both studied QICs were initiated by the same nationwide program in order to increase the use of NQR in Sweden, their characteristics differed widely. The SwedeHF-QIC project ran for 1 year with eight learning sessions compared to the BOA-QIC project with 6 months project time and two learning sessions and one-fifth of the costs. Twice as many improvement activities were started by teams from the SwedeHF-QIC compared to the BOA-QIC but improvements in outcomes varied across the teams, across the different outcomes and along the time period of 4 years. Common activities for both QICs ranged from increased availability and structured follow-up, improved guidelines and care processes as well as better information for patients and colleagues and better dialogue between them. Activities focusing on adherence to standard care programs as well as on increased follow-up of patients seemed to lead to more long-lasting outcome improvements. Independent of the QIC intensity, participants appreciated the programs and perceived that they contributed to their own learning.

In the literature scan by de Silva et al., authors concluded that is it not possible to acknowledge one QIC approach as more effective than another, because the studies compare outcomes of varying models [1]. Furthermore, the scan also highlighted that the approach, structure and activities varied widely in QICs, which is confirmed by the present study. Although, in comparison to the BOA the SwedeHF-QIC program cost was five times higher, included more experts, the project time was twice as long and there were four times as many learning seminars, no clear differences in the number of improvements of the chosen goal indicators could be identified between the SwedeHF and the BOA. In the systematic review of Wells et al., the authors found that the QICs with the most success were those that addressed straightforward aspects of care and those aspects that had a clear gap in evidence-based practice [10]. While the goal indicators of the BOA-QIC were less straightforward, the goal indicators of the SwedeHF were straightforward with some teams who had a clear gap in best practice, for example ‘patients with RAS and beta blocker treatment’ which improved continuously. But those who already performed well on that indicator at the beginning of QIC could not improve more. However, a study of another QIC ‘the Quality Improvement in Coronary Care (QUICC) Study Group’, which used the Swedish Quality Registry for acute coronary care (RIKS-HIA), showed sustained improvements for one and a half years after the QIC. The authors concluded that identification of the most important areas in need of improvement is crucial, as well as not covering too many measures [30]. One essential element of the RIKS-HIA QIC had been training of the teams in ‘how to generate real-time performance feedback and how to use the registry to improve their care processes’ [30]. The importance of data feedback for QI was already mentioned in 1996 by the pioneering QIC of the Northern New England Cardiovascular Disease Study Group [31]. Moreover, in a recent study of 132 healthcare improvement projects in Norway, Brandrud et al. showed that measurement and statistical expertise increased the success of continual improvements [32]. A measurement system and high-quality data registries were also found by Luckenbaugh et al. to be elements of successful QICs, and they suggested automated data acquisition models, as already used by the AQUA registry or other institutional data warehouses, in order to decrease the resource intensive process of data entry and extraction [33]. Furthermore, findings from a recent systematic review confirmed that regular follow-up supported by tailored audits and feedback was important for successful organisational change [34]. However, Nadeem et al. identified in most of the studies in their review a lack of specific information about how local data were used to inform QI [8]. The results of our study revealed that data were not used regularly during ongoing QICs but only at the beginning, and in case of the SwedeHF-QIC, at the end of the project time as well. The use of NQR data as the source for indicator measurement and feedback was experienced to be difficult because of the limited functionality of the registry systems and restricted timeliness of data (some units still filled in registry forms on paper and later entered the data into the registry). Despite the fact that ongoing measurement and data feedback is a given part of QICs [1, 32, 35], it dropped away in our QICs, maybe reasoned in the fact that registries data feedback functionality was limited.

Another reason that data was not reviewed and used continuously during the QIC project time might be associated with the type of goal indicator chosen. As Wells et al. and Carlhed et al. identified, QIC should focus on only a few measures and areas that are in crucial need of improvement [10, 30]. The latter might be the case in the SwedeHF-QIC which might have blurred the focus on improving each indicator. Within the BOA-QIC, the goals were to decrease the average age of registered patients and increase patients’ minimal level of physical activity after 1 year. The first goal was to be reached by acquiring increased knowledge about osteoarthritis treatment among colleagues and orthopaedists. However, an increased number of younger patients is hardly a linear effect by clinicians’ behaviour, but is also dependent on external factors such as the health condition of patients living in the region. The second goal, to increase patients’ physical activity level, was patient-reported (patient level outcome). This indicator may be influenced by their physical activity at onset, their age, comorbidities and the number of patients included in the intervention. Furthermore, the time aspect of the goal indicator (after one year) was beyond the time span of the QIC project (6 months) itself. With this in mind, the BOA-QIC did not focus on audits and data follow-up from the registry. Compared to the BOA sub-goals, all the SwedeHF sub-goals were outcomes that could be directly influenced by behavioural change of the clinicians. Teams that had several activities focusing on increased information about diagnosis and treatment as well as better availability and follow-up (including physical activity) could show continuous improvements along 3 years regarding patients with organised physical activity (team 1 and 5).

As identified by several studies and discussed above, real-time feedback, knowledge of measurement over time and skills in data reviewing might have substantial impacts on continuous improvement efforts beyond the time frame of QICs as a new behaviour is integrated in regular clinical work. This can also be confirmed by the results from Algurén et al. that revealed significant differences in the routine use of SwedeHF and Swedeheart, another Swedish cardiovascular registry with several sub-registries [21]. Swedeheart including RIKS-HIA was used on average 14 times a year for producing healthcare activity statistics, comparing results and reporting them to colleagues, as well as identifying areas for QI work and evaluation of such work, while the SwedeHF was used twice a year, mainly for producing healthcare statistics [21]. The above-named training in the QUICC project in 2004 might still have had impact in 2016 and onwards, through the way using the registry as feedback and improvement system was learnt to be incorporated in clinicians’ daily work. This phenomenon is in line with the findings from the Cochrane review about the effect of audits and feedback on professional practice and healthcare outcomes [36]. The authors concluded that if data feedback is provided continuously in verbal and written formats from a colleague or a supervisor and if it is focused on explicit goals with low baseline performance (but even includes an action plan), it is more effective. However, the effect size varies, depending on what clinical behaviour is targeted by the intervention [37]. As discussed above, the targets of the present QICs were not affected in the same way by clinicians’ behaviours. The diverse results of the teams might also be explained by the fact that there was no proposed action plan by the QIC experts; instead, each team had to find solutions and activities to reach their goals by using QI methods like the PDSA cycles. Out of the 73 identified experts’ recommendations for implementing change (ERIC) strategies by Powell et al., QICs naturally embedded some of them for example; assessment for readiness (organisational commitment), promotion of network weaving and ongoing consultation, time for team meetings, conducting cyclical small tests of change (PDSA cycle) and capturing and sharing local knowledge (done by the learning seminars) [38]. Other strategies such as audits and feedback as well as use of data experts were also included since registry data had to function as the QICs’ quality monitoring system but registries’ functionality was not yet ready for that. Further ERIC strategies such as tailored activities and the development and distribution of educational material were implemented through each teams’ initiatives and accomplished activities. While a common problem of implementation strategies is a misfit of the interventions to the particular context in which they are established [39–41], the QIC teams of the present study developed the interventions on their own in order to reach their goals, and thus interventions were highly adapted to their context.

### Limitations

One might argue that the BOA-QIC does not meet all the requirements of a QIC. For example, several teams sent only one person to each learning session. Conversely, learning within and between teams is an essential goal of QIC structures. However, the final BOA project report revealed that learning happened between the staff and between the teams since QIC teams were established at the home unit instead of within the planned QIC structure. Furthermore, in some QIC teams, learning was facilitated between the BOA-QIC teams and staff that were not included [29]. Findings from the literature describe a large variation of components within the studied QIC [8, 10]. De Silva identified that QICs can range from the structured Breakthrough Series model to approaches that ‘may be less structured ‘communities of practice’ where organisations do not necessarily all focus on the same topic area or which have a less systematic program of learning activities’ [1] (p. 23). From this point of view, the BOA-QIC can be identified as a QIC.

To ensure the accuracy of the presentation of the two structured QICs in Table 1, this table was sent to the leaders of the QICs for validation. The first author also attended the final learning seminar of the SwedeHF-QIC and took part in the teams’ poster presentations in order to ensure data accuracy. The data collection in the NQRs was made in autumn 2017, and since there is a delay in the reporting into the registries, the range of data was set to 2013–2016. Thus, it is probable that all relevant data were retrieved. To ensure the correctness of interpretation of the data and to find the most accurate way to present it, the data were discussed with staff working at the registries’ administrative offices.

## Conclusions

The results of the present study indicate that there is no linear relationship between QIC designs and sustained improvements on outcomes. The results confirm earlier studies that there exists a broad variation of designing and driving collaboratives. However, by opening the black box and looking closer into the activities performed by the QIC teams, it was clear that the longer the QIC, the more QI activities were performed. Activities focusing on adherence to standard care programmes as well as on increased follow-up of patients seemed to lead to more long-lasting improvements. Yet outcomes as specific measurable aspects of care in need of improvement should be chosen carefully and the focus should be on a few aspects where a clear gap in evidence-best-practice exists. Although identified in earlier studies that data follow-up and measurement skills as well as well-functioning data warehouses contribute to sustained improvements, the present registries’ functionality and QICs at this time did not support those aspects sufficiently, which might impede long-lasting improvements. Further studies on QICs and their impact on improvements beyond the project time should investigate more closely behaviour changes in using audits, and data feedback systems in particular.

## Data Availability

The datasets used and analysed during the current study are available from the corresponding author. The QIC project reports are public documents and can also be obtained from the respective registry centre. The registry data can also be obtained from the contact person of each registry.

## Abbreviations

BOA: Better Management of Patients with OsteoArthritis Registry
BOA-QIC: Quality Improvement Collaborative using the BOA in order to improve care
ERIC: Experts’ recommendations for implementing change
NQRs: Swedish National Quality Registries
PDSA: Plan-Do-Study-Act
QI: Quality improvement
QIC: Quality improvement collaborative
QUICC: Quality improvement in coronary care
RAS: Renin-angiotensin system
RIKS-HIA: Swedish Register of Information and Knowledge about Swedish Heart Intensive Care Admission
SALAR: Swedish Association of Local Authorities and Regions
SOASP: Supported Osteoarthritis Self-Management Programme
SwedeHF: Swedish Heart Failure Registry
SwedeHF-QIC: Quality Improvement Collaborative using the SwedeHF in order to improve care
